# A Highly Automated Computational Method for Modeling of Intracranial Aneurysm Hemodynamics

**DOI:** 10.3389/fphys.2018.00681

**Published:** 2018-06-12

**Authors:** Jung-Hee Seo, Parastou Eslami, Justin Caplan, Rafael J. Tamargo, Rajat Mittal

**Affiliations:** ^1^Department of Mechanical Engineering, Johns Hopkins University, Baltimore, MD, United States; ^2^Department of Neurosurgery, Johns Hopkins Medicine, Baltimore, MD, United States

**Keywords:** cerebral aneurysm, hemodynamics, computational fluid dynamics, immersed boundary method, automatic segmentation

## Abstract

Intracranial aneurysms manifest in a vast variety of morphologies and their growth and rupture risk are subject to patient-specific conditions that are coupled with complex, non-linear effects of hemodynamics. Thus, studies that attempt to understand and correlate rupture risk to aneurysm morphology have to incorporate hemodynamics, and at the same time, address a large enough sample size so as to produce reliable statistical correlations. In order to perform accurate hemodynamic simulations for a large number of aneurysm cases, automated methods to convert medical imaging data to simulation-ready configuration with minimal (or no) human intervention are required. In the present study, we develop a highly-automated method based on the immersed boundary method framework to construct computational models from medical imaging data which is the key idea is the direct use of voxelized contrast information from the 3D angiograms to construct a level-set based computational “mask” for the hemodynamic simulation. Appropriate boundary conditions are provided to the mask and the dynamics of blood flow inside the vessels and aneurysm is simulated by solving the Navier-Stokes equations on the Cartesian grid using the sharp-interface immersed boundary method. The present method does not require body conformal surface/volume mesh generation or other intervention for model clean-up. The viability of the proposed method is demonstrated for a number of distinct aneurysms derived from actual, patient-specific data.

## Introduction

An aneurysm is a pathological, localized, balloon-like bulge in the wall of a blood vessel. Although aneurysms can occur in any vessel, intracranial aneurysm (ICA) (or cerebral aneurysm) and abdominal aortic aneurysm (AAA) are most common and clinically significant. Intracranial aneurysms can present incidentally (i.e., unruptured) or may present in the form of aneurysmal subarachnoid hemorrhage (aSAH) following intradural rupture. The overall incidence of aSAH in the Western world is 6–8 per 100,000 people per year (Zacharia et al., 2010) and mortality rates from aSAH are nearly 50%. Of the patients who do survive, less than 60% will return to a neurologic baseline allowing them to function independently (Zacharia et al., 2010). Given the complexity of treatment, and the care required for survivors with devastating neurologic injury, the cost of aSAH is staggering.

Aneurysms considered to be at high risk of rupture are usually treated by surgical intervention such as clipping of the aneurysm itself or implementation of a prosthetic graft to prevent rupture. Such surgical interventions, however, bring their own risks such as bleeding, stroke, and vessel spasms (Raaymakers et al., 1998; Tomasello et al., 1998). Introduction of rupture-prevention devices (e.g., stent graft) can cause thrombosis and increase thrombo-embolic risk (International Study of Unruptured Intracranial Aneurysms Investigators, 1998; Song et al., 2004). Thus, prompt and accurate stratification of risk is the key to making sound clinical decisions about surgical intervention. Significant hurdles however exist in developing accurate risk stratification metrics that are grounded in the biomechanics of aneurysm growth and these have stymied not only our ability to understand aneurysm growth, but also, effective clinical interventions for this devastating condition.

Aneurysms manifest in a vast variety of morphologies (shapes, sizes, orientations and locations) and their growth is also subject to patient-specific conditions of age, gender, flow rate, blood pressure, heart rate, etc. Thus, any statistical correlation that is used for risk stratification should be based on large, likely, O(10^4^) sample size that can cover the vast parameter space associated with aneurysms and generate reliable statistical correlations. While patient specific morphology and conditions are the primary determinants of growth and rupture risk, the connection between these factors and risk is highly complex due to the intervening non-linear effects of hemodynamics and vessel wall structural dynamics. Thus, current clinical guidelines for aneurysm treatment, which are based primarily on morphology (e.g., aneurysm diameter, Desai et al., 2010), have low sensitivity and specificity (Juvela et al., 1993; International Study of Unruptured Intracranial Aneurysms Investigators, 1998; Wiebers, 2003; Desai et al., 2010). Risk stratification approaches that go beyond morphology, and incorporate biomechanics could transform the treatment of aneurysms.

Physics-based computational models of aneurysm biomechanics (Cebral et al., 2005a; Valencia et al., 2008; Castro et al., 2009; i.e., hemodynamics and/or structural mechanics) hold great promise in this context. In particular, hemodynamics is essential to the estimation of aneurysm rupture risk not only because hemodynamics is the key intermediary between morphology and vessel wall mechanics but also because hemodynamic metrics are very sensitive to the geometrical and flow conditions (Cebral et al., 2005b; Valencia et al., 2013). Fortunately, modern imaging modalities [Computational Tomography Angiography (CTA) and 3D Rotating Angiogram (3DRA)] provide inputs that are suitable and generally sufficient for computational fluid dynamics modeling. The primary limitation of the current approaches however is that they are not designed to scale to large sample sizes that are necessary for developing insights and reliable statistical correlations/metrics.

In order to perform hemodynamic simulations for large number of aneurysm cases, pipe-lined (Cebral et al., 2005a) and automated methods to convert medical imaging data to simulation-ready configuration with minimal (or no) human intervention are required. Currently, most simulations of aneurysm hemodynamic are performed with the finite-volume or finite-element methods (Shojima et al., 2004; Cebral et al., 2005b, 2015; Valencia et al., 2008, 2013; Castro et al., 2009; McGah et al., 2014; Valen-Sendstad and Steinman, 2014) that require surface and volume meshes. Commercial CFD software based on the finite volume/element method are also often employed (Meng et al., 2006; McGah et al., 2014; Valen-Sendstad and Steinman, 2014), for which the segmented vessel/aneurysm geometry and surface/volume meshes need to be provided. Most of the current segmentation and simulation methods that involve surface and volume mesh generations necessitate substantial human intervention. The traditional approach consists of the following steps; (i) segmentation of lumen from the angiogram data, (ii) 3D model generation, (iii) cleaning-up and truncation of the model (e.g., cutting out the vessels outside the region of interest), (iv) surface mesh generation, and (v) volume mesh generation. An open source or commercial software can be employed for each step, but it still requires substantial human intervention to interface each step and determine the parameters. Thus, the traditional approach may not be adequate to deal with large number of individual cases envisioned here. Furthermore, conventional computational fluid dynamics simulation methodologies can be quite sensitive to the quality of the segmentation and grid generation, and this may necessitate attention to the quality of the segmented geometry and computational grid (Valen-Sendstad and Steinman, 2014). In the present study, a highly-automated method based on the immersed boundary method (Mittal and Iaccarino, 2005) framework is proposed to construct computational models directly and rapidly from medical imaging data. The key idea is the direct use of voxelized contrast information from the 3D angiograms to construct a level-set based computational “mask” for the simulation. In this way, 3D, simulation-ready models of the vessel of interest can be constructed automatically and rapidly, and no body-conformal grids (surface and volume) need to be generated for the flow simulation.

An immersed boundary method based on the “masking function” on the Cartesian grid has previously been applied to the aneurysm hemodynamics (Mikhal and Geurts, 2014), but the method employed a simple volume penalization, and the geometry was represented by set of Cartesian voxels. Better representation of the aneurysm/vessel geometry on the Cartesian grid can be achieved by using a level-set function. The level-set function based methods have been used for the simulation of aneurysm hemodynamics on the Cartesian grid using a lattice Boltzmann method (LBM) (He et al., 2009; Závodszky and Paál, 2013) and a boundary data immersion method (Otani et al., 2018). The latter method, however, still employed a surface mesh to construct the level-set function. In the present study, we employ the masking function for the automatic segmentation of vessel/aneurysm from the medical imaging data. The vessel/aneurysm boundaries are then represented by the level-set function constructed directly by using the contrast information. A previously developed and validated hemodynamic flow solver based on the sharp-interface immersed boundary method (Mittal et al., 2008) is adopted for modeling aneurysm hemodynamics on the Cartesian grid. In this paper, we report the key components of the highly automated simulation procedure using the immersed boundary method such as a 3D, region-growing technique for automatic vessel segmentation and cleaning, a level-set based, immersed boundary flow simulation module, and a suitable method for post-processing the data. The present method has been tested with small sample set of patient-data.

## Materials and method

### Procedure of highly-automated hemodynamic modeling

The overall procedure of the highly-automated hemodynamic modeling using the 3D angiogram data is the following:

1. For a given angiogram of the vasculature around a cerebral aneurysm, a user specifies the subset of the 3D angiogram for the region of interest (ROI) around the target aneurysm, and identifies the inflow vessel. The user also set a seed point for the region growing operation and a threshold contrast intensity (I_0_) for the identification of the lumen. The inflow condition (flowrate and heart rate) can also be set by the user, if patient-specific information is available. These are the only manual operations required for the present method.
2. The region growing operation is performed from the seed point, which identifies the lumen region in the ROI and generates a masking function (M) with value of 1 in the lumen and 0 otherwise.
3. A Cartesian grid is generated automatically in the ROI, and a level-set function (ϕ) is defined by the contrast intensity and the given threshold value. The level-set function is used to find the location of the lumen wall.
4. A vessel centerline is identified automatically by using the masking function and the inflow spatial velocity profile is prescribed per the given flow condition.
5. Flow simulation is performed on the Cartesian grid. The flow equations are only solved for the lumen region using the masking function, and the wall boundary condition is applied by using the level-set function.
6. As a post-processing, wall shear stresses and other metrics are calculated.

Each of these steps are described in detail in the following sections.

### Region of interest and boundary conditions

**Figure 1:**
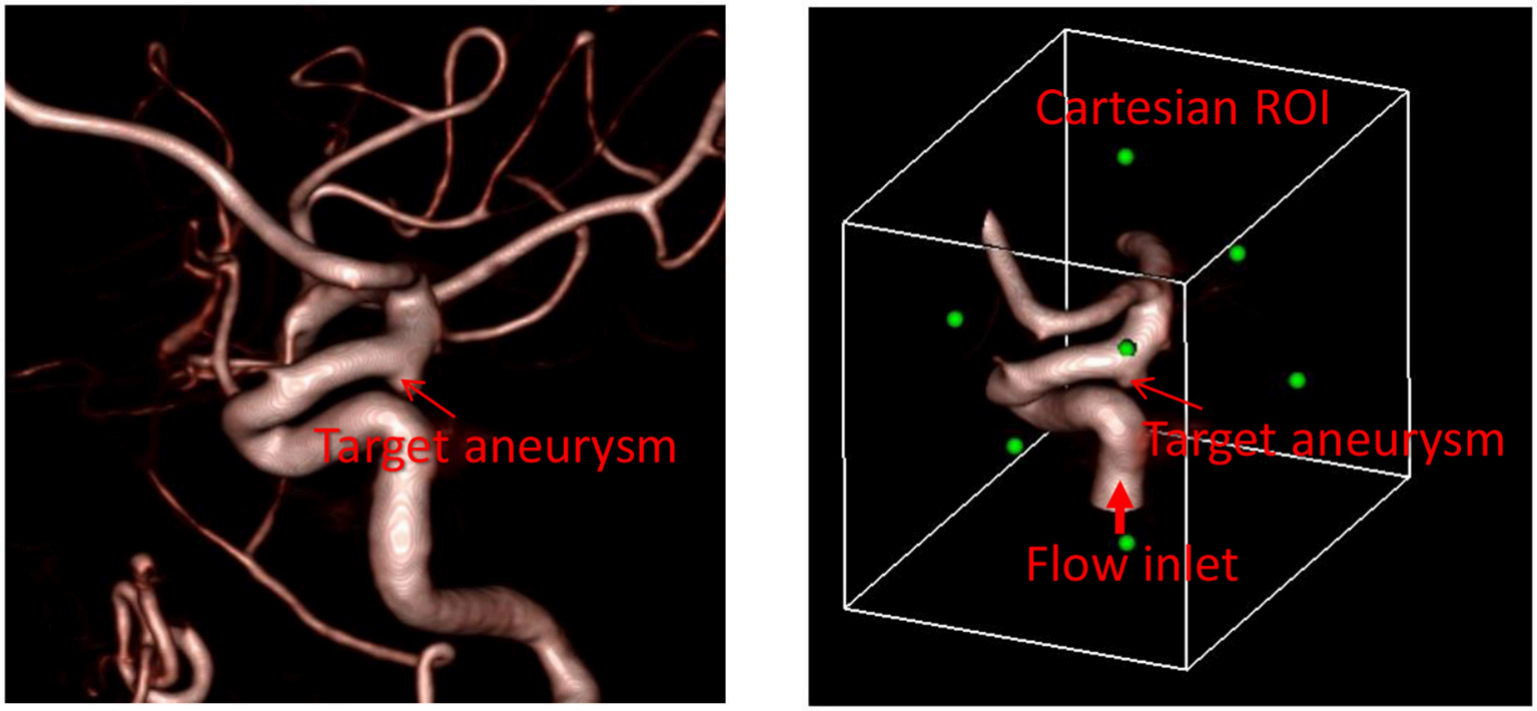
Preparation of angiogram data for the automated flow simulation. **(Left)** 3D angiogram of vessels with a target aneurysm identified. **(Right)** Cartesian ROI set around the target aneurysm with the inflow vessel identified. Green dots represent the center of each face of the Cartesian ROI.

The first step required for the hemodynamic modeling is to specify the region of interest (ROI) around the target aneurysm. By visualizing the angiogram, the user should identify the target aneurysm of interest and set the Cartesian ROI around it (see Figure 1). This can be done by specifying the range of spatial indices (i, j, k) for the Cartesian ROI domain, for example, i_min_ ≤ i ≤ i_max_, j_min_ ≤ j ≤ j_max_, and k_min_ ≤ k ≤ k_max_. Once the ROI is set, there could be number of vessels that intersect with the ROI boundaries. Boundaries of flow domain can easily be identified during the automatic segmentation phase using the spatial index of Cartesian ROI domain. For example, the voxels masked for the flow domain at the Cartesian ROI boundaries (i_min_, i_max_, j_min_, j_max_, k_min_, k_max_) generate the boundaries of the flow domain. The flow direction (inflow or outflow) in each of these intersecting vessels needs to be identified. For inflow vessels, the user can specify the flow rate and/or flowrate wave form if available. The user also sets a seed point in the lumen region which is connected to the target aneurysm and a threshold contrast intensity (I_0_) for the region growing operation.

### Automatic segmentation and cleaning

**Figure 2:**
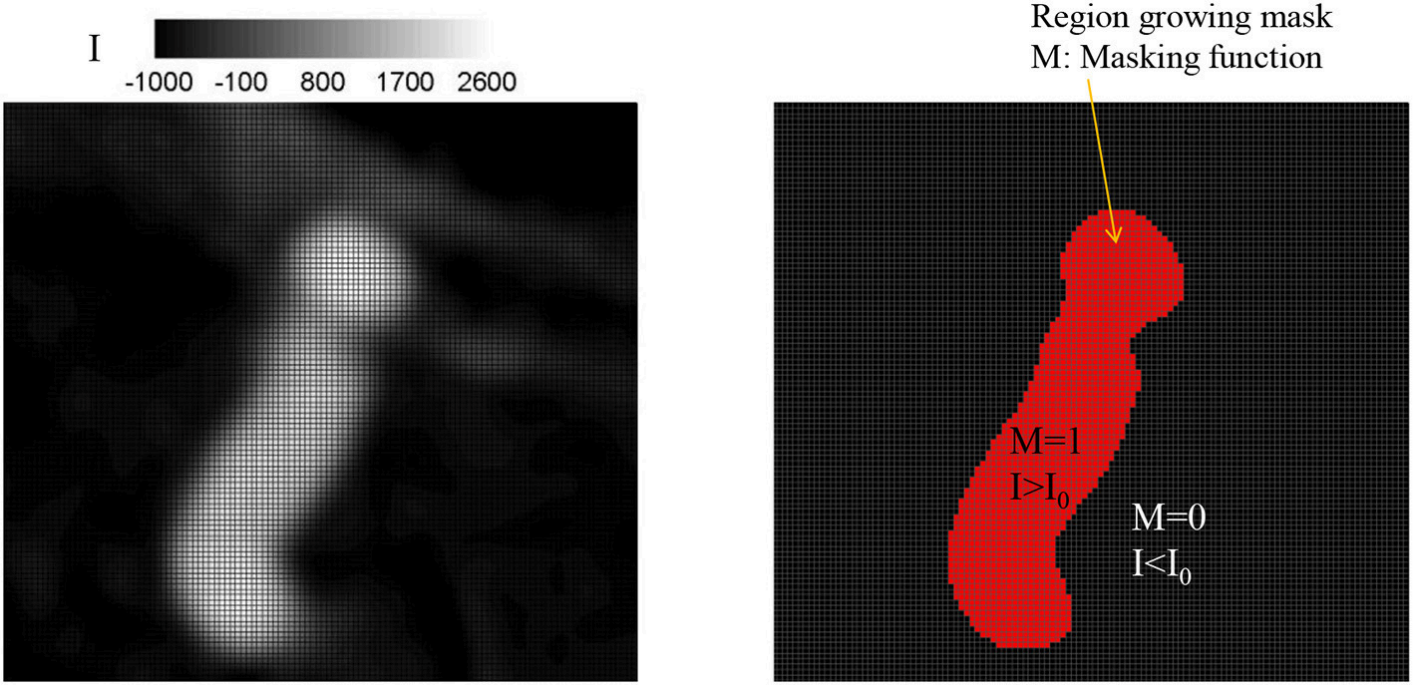
Identifying lumen region by a region growing algorithm. **(Left)** Original grayscale image with the intensity (I). **(Right)** Identified lumen with the threshold intensity, I_0_ = 500. The region for which intensity, I, greater than the threshold, I_0_ is identified as lumen and the masking function value M is set to 1; otherwise M = 0.

Once the seed point and the threshold intensity (I_0_) are set, the 3D region growing operation is performed to automatically segment the lumen of interest and perform clean-up. The region growing runs on the Cartesian voxel space of the 3D angiogram data and each voxel of the imaging data serves as a Cartesian fluid cell. Starting from the seed point, the edge cells grow to neighboring Cartesian cells if the intensity of the cell (I) satisfies the criteria, I>I_0_. Additional criteria based on the gradient of intensity, e.g., ΔI < ΔI_max_ can also be employed. The choice of threshold can affect the size of the segmented vessel/aneurysm, and subsequently, the simulation results. The user may reset the threshold by checking the morphology of the segmented vessel/aneurysm. For this reason, the threshold value may need to be chosen by a trained expert. The mask function (M) is set to 1 for the growing region and 0 otherwise (see Figure 2). The process continues until no further growth is possible, and the connected lumen region is segmented based on the masking function (*M* = 1). The flow simulation is performed only for the volume where *M* = 1, and thus the other region where *M* = 0 including lumen volumes that are not connected to the target aneurysm is automatically cleaned-up. A key element in the present method is that, unlike other conventional body-conformal numerical methods (finite-difference, finite-volume, or finite-element) that are commonly used in hemodynamic simulations (Soto et al., 2004; Updegrove et al., 2017), no surface mesh is generated for the segmented lumen. This alleviates complexities associated with mesh quality, and enhances the automation and robustness of the simulation tool.

### Level-set function and wall boundary condition

The Cartesian grid based on the voxel space of the 3D angiogram also serve as the grid for the flow simulation. In order to define the lumen wall boundary which is not conformal to the Cartesian grid, a level-set function, ϕ is defined by using the intensity (I) information:

(1) $$\begin{array}{r} \phi_{0}\left(\text{i},\text{j},\text{k}\right)=\text{I}\left(\text{i},\text{j},\text{k}\right)-{\text{I}}_{0}, \end{array}$$

where (i, j, k) are grid indices. If the angiogram is noisy, a low-pass, spatial filtering can be employed as a preprocessing step to smooth out the level set function, ϕ. A filtering scheme for 3 × 3 × 3 stencils is given by

(2) $$\begin{array}{rcl} \text{ϕ}\left(\text{i},\text{j},\text{k}\right) & = & \overset{1}{\underset{\text{p}=-1}{\sum }}\overset{1}{\underset{\text{q}=-1}{\sum }}\overset{1}{\underset{\text{r}=-1}{\sum }}{\left(\frac{1}{2}\right)}^{3}{\left(\frac{1}{2}\right)}^{\left(\left|\text{p}|+|\text{q}|+|\text{r}\right|\right)} \end{array}$$

$$\begin{array}{r} \phi_{0}\left(\text{i}+\text{p},\text{j}+\text{q},\text{k}+\text{r}\right). \end{array}$$

**Figure 3:**
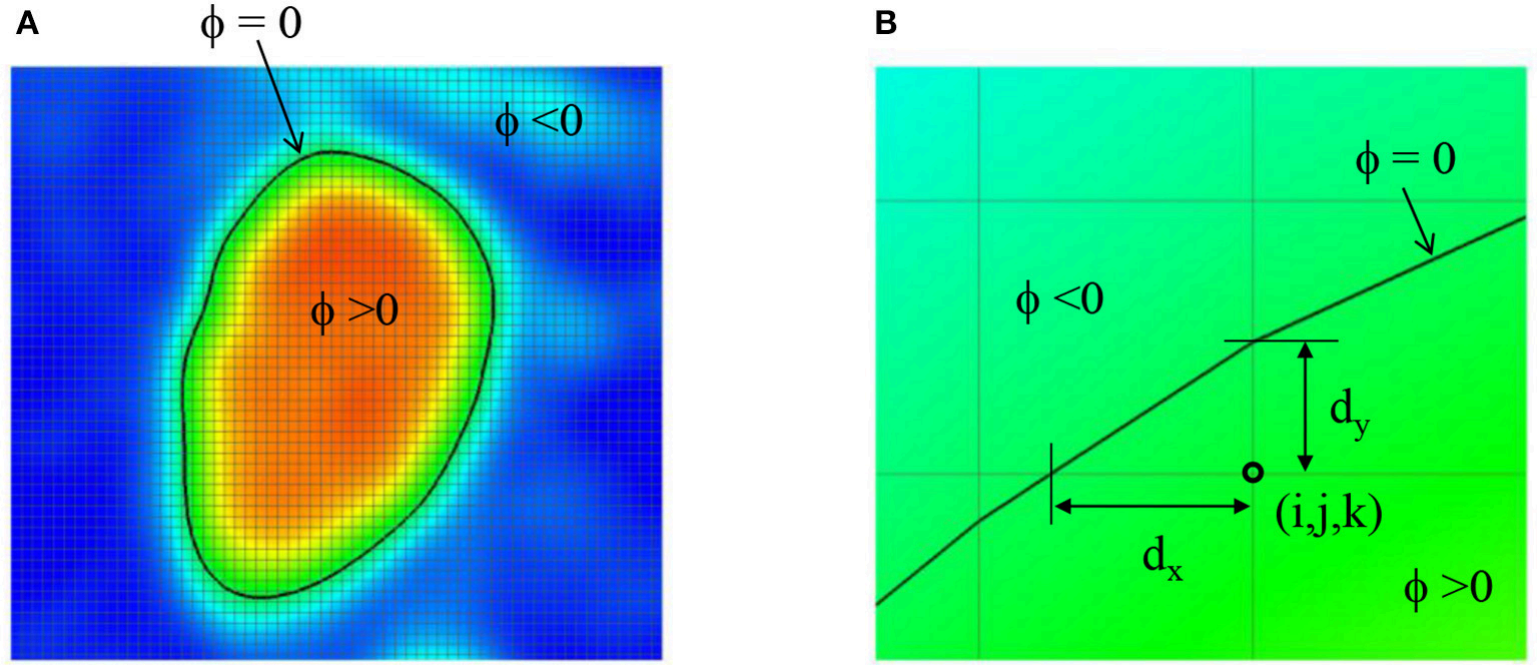
**(A)** Level-set function ϕ defined by the image intensity. The lumen wall surface is defined by ϕ = 0. **(B)** Distance from the Cartesian grid point to the lumen wall location.

**Figure 4:**
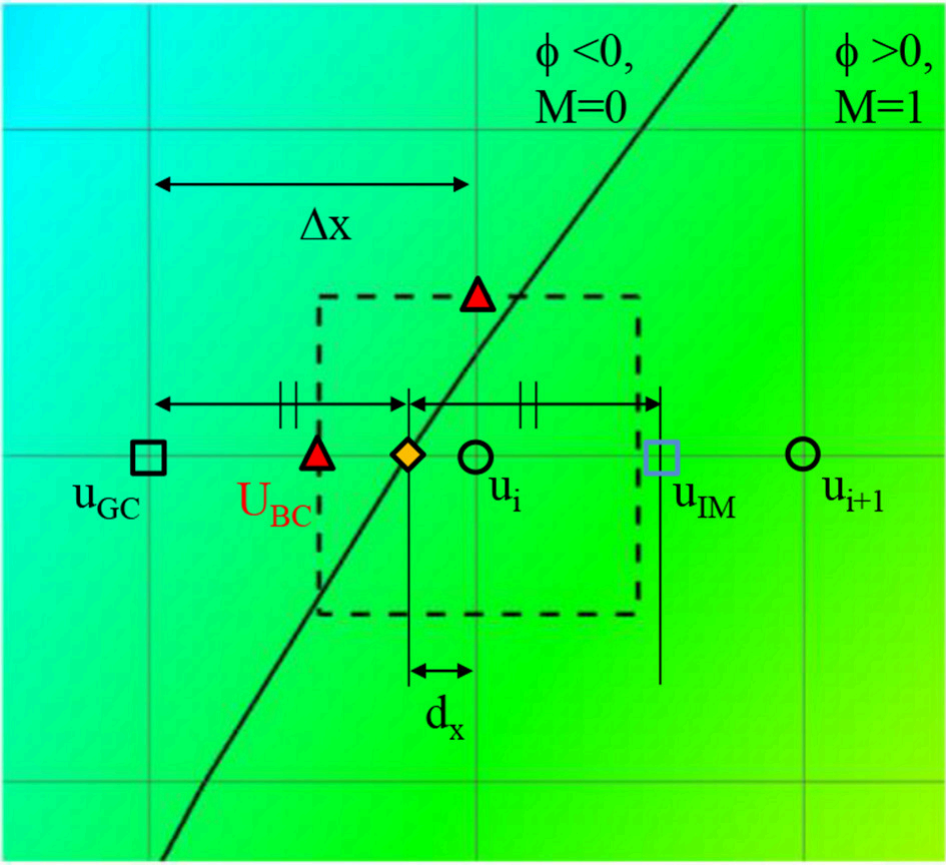
A schematic describing the imposition of wall boundary condition. The rectangle with dashed line represents a computational cell. Triangle symbol indicates the cell face center where the boundary velocity, U_BC_ is imposed. Detailed description is provided in the text.

The lumen wall surface is defined by ϕ = 0, and ϕ > 0 for the hemodynamic flow region (Figure 3A). To apply the boundary condition on the lumen wall, the distance from the Cartesian grid point to the wall location is required and this is computed automatically as shown in Figure 3B by

(3) $$\begin{array}{r} {\text{d}}_{\text{x}}=\frac{\phi }{\partial \phi /\partial \text{x}},{\text{d}}_{\text{y}}=\frac{\phi }{\partial \phi /\partial \text{y}},{\text{d}}_{\text{z}}=\frac{\phi }{\partial \phi /\partial \text{z}}, \end{array}$$

where d_x_, d_y_, and d_z_ are the distances to the wall in x, y, and z directions, respectively. Once these distances are calculated, the wall boundary condition for the flow simulation is imposed by the following way. Since the flow equations are solved on the Cartesian grid, the boundary conditions for the flow velocities are applied by imposing the cell face velocity, U_BC_ as shown in Figure 4. The value of U_BC_ is obtained by interpolation/extrapolation with the velocity on the wall u_w_, and on the flow region, u_i_. In the x-direction, for example, for the no-slip, stationary wall (u_w_ = 0), if the distance from the Cartesian grid point to the wall, d_x_, is smaller than the half of grid spacing, Δx/2, U_BC_ is given by the linear interpolation:

(4) $$\begin{array}{r} {\text{U}}_{\text{BC}}=u_{\text{i}}\left(1-\frac{\Delta \text{x}}{2d_{\text{x}}}\right). \end{array}$$

If d_x_>Δx/2, U_BC_ is calculated by a ghost fluid method (Fedkiw et al., 1999). First, the adjacent grid point outside the flow region is identified and marked as a ghost point. To find the velocity on the ghost point, u_GC_, the image point in the flow region is found by mirroring the ghost point with respect to the wall position. Note that the distance from the ghost point to the wall is the same with the distance from the wall to the image point. For the no-slip, stationary wall (u_w_ = 0), therefore, u_GC_ is given by

(5) $$\begin{array}{r} {\text{u}}_{\text{GC}}=-{\text{u}}_{\text{IM}}, \end{array}$$

where u_IM_ is the velocity on the image point, which can be found by interpolation using flow velocities on the Cartesian grid points;

(6) $$\begin{array}{r} {\text{u}}_{\text{IM}}={\text{u}}_{\text{i}}+\frac{{\text{u}}_{\text{i}+1}-{\text{u}}_{\text{i}}}{\Delta \text{x}}\left(\Delta \text{x}-2{\text{d}}_{\text{x}}\right). \end{array}$$

Finally, U_BC_ is given by

(7) $$\begin{array}{r} {\text{U}}_{\text{BC}}=\frac{1}{2}\left({\text{u}}_{\text{GC}}+{\text{u}}_{\text{i}}\right)=\frac{{\text{u}}_{\text{i}}-{\text{u}}_{\text{i}+1}}{\Delta \text{x}}\left(\frac{\Delta \text{x}}{2}-{\text{d}}_{\text{x}}\right). \end{array}$$

### Inflow velocity profile

The flow simulations in the proposed method are performed by imposing the flow velocity in the inflow vessels. It is assumed that the inflow velocity is aligned with the vessel centerline. Also, the radial distribution of the velocity profile is in general specified as a combination of a steady parabolic and an oscillatory Womersley profile as follows

(8) $$\begin{array}{r} \text{u}\left(\text{r},\text{t}\right)={\text{u}}_{\text{parabolic}}\left(\text{r}\right)+u_{\text{oscillatory}}\left(\text{r},\text{t};\text{Wo}\right). \end{array}$$

where the steady flow profile is prescribed as ${\text{u}}_{\text{parabolic}}\left(r\right)={\text{U}}_{0}\left(1-{\text{r}}^{2}/{\text{R}}^{2}\right)$ and the oscillatory profile u_oscillatory_ is determined in terms of the Womersley number $\text{Wo}=\text{R}\sqrt{\rho 2\pi {\text{f}}_{0}/\mu }$, (Loudon and Tordesillas, 1998) where f_0_ is the heart rate (Hz), R is the radius of the vessel and μ is the Newtonian viscosity of blood. The oscillatory profile can also be constructed by superposing a number of Fourier modes at harmonics of f_0_ to model more realistic inflow profiles (Cebral et al., 2005a; Valencia et al., 2008). The radius of the artery is available directly from the segmentation. The other parameters needed are U_0_ and the heart rate f_0_. Both of these may either be provided by the user based on patient-data, or in the absence of this information, simulations may be carried out for a range of these parameters.

**Figure 5:**
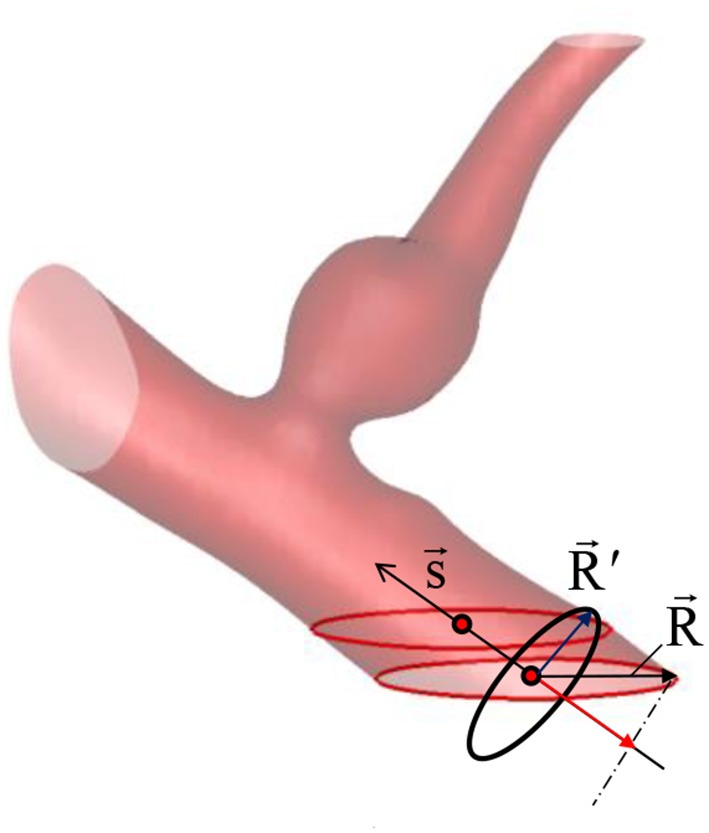
Vessel center line ($\vec{\text{s}}$) and radius ($\vec{{\text{R}}^{\prime }}$) vectors. The vessel/aneurysm geometry is automatically segmented in the Cartesian ROI. The center line vector is defined by the vessel center points (red circle symbols) and the vessel radius vector is calculated from the in-plane radius vector ($\vec{\text{R}}$).

Because the inflow vessels are not always normal to the boundary of the ROI, the following prescription is applied. First, the local, unit vessel centerline vector, $\vec{\text{s}}$ is determined by calculating the vessel center point, ${\vec{\text{x}}}_{\text{c}}$ using the masking function, M(i, j, k). For example, if the inflow boundary is at the ROI boundary, k = k_min_, the vessel center points are calculated at each k index near the boundary by;

(9) $$\begin{array}{r} {\vec{\text{x}}}_{\text{c}}\left(k\right)=\frac{\overset{{\text{i}}_{\max}}{\underset{\text{i}={\text{i}}_{\min}}{\sum }}\overset{{\text{j}}_{\max}}{\underset{\text{j}={\text{j}}_{\min}}{\sum }}\text{M}\left(\text{i},\text{j},\text{k}\right)\cdot \;\vec{\text{x}}\left(\text{i},\text{j},\text{k}\right)}{\overset{{\text{i}}_{\max}}{\underset{\text{i}={\text{i}}_{\min}}{\sum }}\overset{{\text{j}}_{\max}}{\underset{\text{j}={\text{j}}_{\min}}{\sum }}\text{M}\left(\text{i},\text{j},\text{k}\right)}, \end{array}$$

where $\vec{\text{x}}$ is the grid center coordinates. The local centerline vector, $\vec{\text{s}}$ is then obtained by $\vec{\text{s}}={\vec{\text{x}}}_{\text{c}}\left({\text{k}}_{\min}+\Delta \text{k}\right)-{\vec{\text{x}}}_{\text{c}}\left({\text{k}}_{\min}\right)$. Once the vessel center point is found at the inflow boundary, in-plane radius vector, $\vec{R}$ can be defined on any grid points inside the inflow vessel lumen (see Figure 5) by $\vec{\text{R}}=\vec{\text{x}}-{\vec{\text{x}}}_{\text{c}}$. To prescribe a radial velocity profile, the radius vector normal to the vessel centerline vector is computed by a vector rejection:

(10) $$\begin{array}{r} {\vec{\text{R}}}^{\prime }\left(\text{i},\text{j},\text{k}\right)=\vec{\text{R}}\left(\text{i},\text{j},\text{k}\right)-\left(\vec{\text{R}}\left(\text{i},\text{j},\text{k}\right)\cdot \vec{\text{s}}\right)\vec{\text{s}}. \end{array}$$

The radial inflow velocity profile can be specified using this radius vector. For example, a fully developed, parabolic profile for steady flow is given by

(11) $$\begin{array}{r} \vec{\text{U}}\left(\text{i},\text{j},\text{k}\right)={\text{U}}_{0}\left[1-{\left(\frac{\left|{\vec{\text{R}}}^{\prime }\right|}{{{\text{R}}^{\prime }}_{\max}}\right)}^{2}\right]\vec{\text{s}}, \end{array}$$

where ${{\text{R}}^{\prime }}_{\max}$ is the maximum value of $\left|{\vec{\text{R}}}^{\prime }\right|$ over the inflow boundary. More realistic, time dependent velocity profile can also be employed by using Equation (8).

### Immersed boundary flow solver

The hemodynamic simulation is performed by solving the incompressible Navier-Stokes equations on the Cartesian grid using the immersed boundary method (Mittal and Iaccarino, 2005). In the present study, a Newtonian fluid assumption is employed and the governing equations for the hemodynamic flow are given by

(12) $$\begin{array}{r} \nabla \cdot \vec{\text{U}}=0,\rho \frac{\partial \vec{\text{U}}}{\partial t}+\rho \left(\vec{\text{U}}\cdot \nabla \right)\vec{\text{U}}+\nabla \text{P}=\mu \nabla^{2}\vec{\text{U}}, \end{array}$$

where $\vec{\text{U}}$ is the flow velocity vector, P is the pressure, ρ and μ are the density and dynamic viscosity of the blood. The equations are discretized by using the second-order finite-difference methods in time and space. The flow solver used in this study is a modified version of the immersed boundary, incompressible flow solver, ViCar3D (Mittal et al., 2008). The solver has been extensively validated for a variety of laminar/turbulent flows (Mittal et al., 2008; Vedula et al., 2014), and employed for a wide range of studies of cardiac hemodynamics, including modeling of left ventricular (LV) hemodynamics with natural (Zheng et al., 2012; Seo et al., 2014) and prosthetic mitral valves (Choi et al., 2014), role of ventricular trabeculae on LV hemodynamics (Vedula et al., 2016), and LV thrombus formation (Seo et al., 2016). The solver is also fully parallelized by using a message passing interface (MPI) library, and the performance scales well up to O(1000) processors. As mentioned above, Cartesian voxel space of the 3D angiogram can directly be used as a Cartesian grid for the flow simulation. For 3D angiograms, the voxel size is about 0.2~0.3 mm, and this is adequate as the grid spacing for the flow simulation. The flow equations are solved only for the lumen region identified by the masking function, M, and the lumen wall boundary condition is prescribed by the level-set function method shown in section Level-Set Function and Wall Boundary Condition. The procedure of solving Equation (12) is as follows: the second equation of Equation (12) (momentum equation) is discretized on the Cartesian grid using the second-order finite difference method without the pressure gradient term, and integrated in time using the second-order Crank-Nicolson method to obtain the intermediate velocity fields. Applying the continuity equation (the first equation of Equation 12), one can obtain the Poisson equation for the pressure, and this is solved by using a parallelized bi-conjugate gradient method. Finally, the intermediate velocity field is corrected by adding the pressure gradient term to advance the solution over one time-step. More detailed solution procedure can be found in Mittal et al. (2008).

### Post-processing

**Figure 6:**
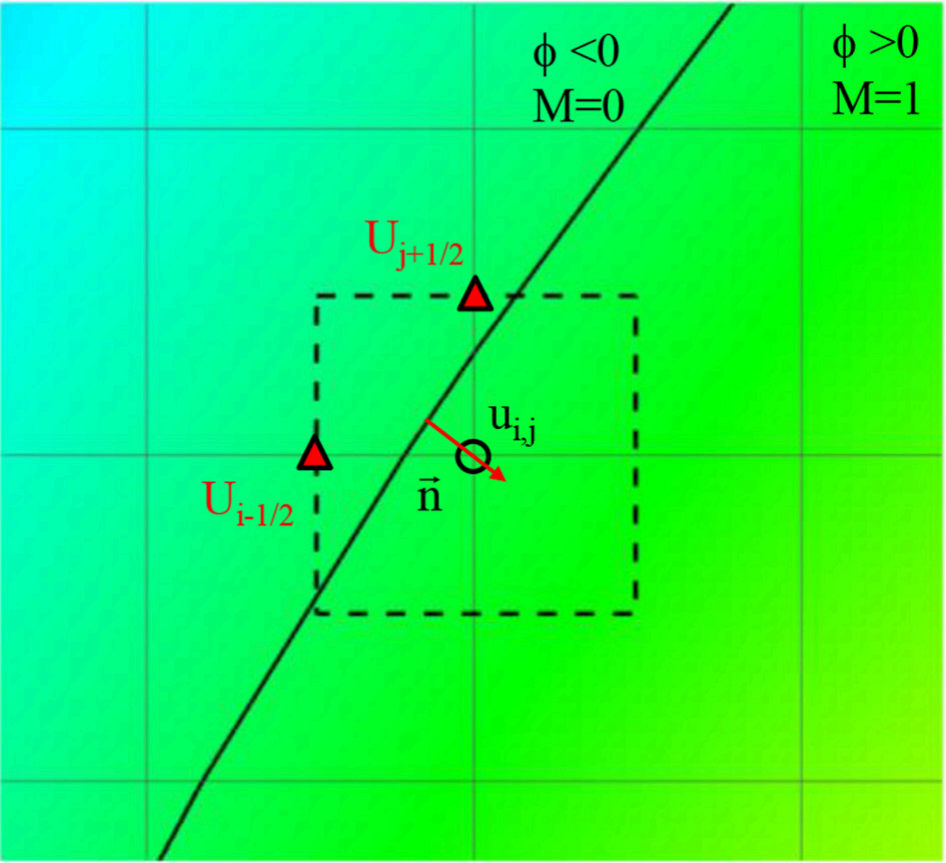
Schematic describing of the calculation of velocity gradient near the wall. Solid line: Vessel wall boundary identified by the level-set function. Rectangle with dashed line: A computational cell adjacent to the wall. Triangle symbol: The cell face center where the boundary velocity is imposed.

Flow-induced forces on the lumen wall (pressure and viscous shear stress) are considered important factor for characterizing the aneurysm rupture risk. Since the pressure gradient in the wall normal direction is usually set to 0 (∂*P*/∂*n* = 0), the pressure on the lumen wall can easily be calculated by simple interpolation using the values on Cartesian fluid cells near the wall. The viscous shear stress involves velocity gradients, and thus is calculated by the following way. On the Cartesian fluid cell adjacent to the wall, the velocity gradients are calculated by using the boundary velocities at the cell faces as shown in Figure 6.

(13) $$\begin{array}{r} \frac{\partial \text{u}}{\partial \text{x}}\approx \frac{{\text{U}}_{\text{i}+1/2}\left(1-{\text{M}}_{\text{i}+1,\text{j},\text{k}}\right)+{\text{U}}_{\text{i}-1/2}\left(1-{\text{M}}_{\text{i}-1,\text{j},\text{k}}\right)+\left({\text{M}}_{\text{i}+1,\text{j},\text{k}}-{\text{M}}_{\text{i}-1,\text{j},\text{k}}\right){\text{u}}_{\text{i},\text{j},\text{k}}}{\Delta \text{x}/2},\\ \frac{\partial \text{u}}{\partial \text{y}}\approx \frac{{\text{U}}_{\text{j}+1/2}\left(1-{\text{M}}_{\text{i},\text{j}+1,\text{k}}\right)+{\text{U}}_{\text{j}-1/2}\left(1-{\text{M}}_{\text{i},\text{j}-1,\text{k}}\right)+\left({\text{M}}_{\text{i},\text{j}+1,\text{k}}-{\text{M}}_{\text{i},\text{j}-1,\text{k}}\right){\text{u}}_{\text{i},\text{j},\text{k}}}{\Delta \text{y}/2},\\ \frac{\partial \text{u}}{\partial \text{z}}\approx \frac{{\text{U}}_{\text{k}+1/2}\left(1-{\text{M}}_{\text{i},\text{j},\text{k}+1}\right)+{\text{U}}_{\text{k}-1/2}\left(1-{\text{M}}_{\text{i},\text{j},\text{k}-1}\right)+\left({\text{M}}_{\text{i},\text{j},\text{k}+1}-{\text{M}}_{\text{i},\text{j},\text{k}-1}\right){\text{u}}_{\text{i},\text{j},\text{k}}}{\Delta \text{z}/2}, \end{array}$$

where Δx, Δy, Δz are the grid spacing, M_i, j, k_ is the masking function value, and subscripts denote grid indices. The wall normal vector is given by the gradient of the level-set function as

(14) $$\begin{array}{r} \vec{\text{n}}=\frac{\nabla \phi }{\left|\nabla \phi \right|}. \end{array}$$

For the incompressible flow, the normal gradient of the wall normal velocity component on the stationary wall is supposed to be zero. It is found however that this is not guaranteed numerically for the present method, because the normal gradient is not directly calculated on the wall. This numerical error scales with the grid spacing (Δx). The viscous wall shear stress is then calculated by using the tangential velocity gradient in the wall normal direction as

(15) $$\begin{array}{r} {\vec{\tau }}_{\text{w}}=\mu \frac{\partial {\vec{\text{u}}}_{\text{t}}}{\partial \text{n}}=\mu \left(\frac{\partial \vec{\text{u}}}{\partial \text{n}}-\frac{\partial {\vec{\text{u}}}_{\text{n}}}{\partial \text{n}}\right)=\mu \left\{\nabla \vec{\text{u}}\cdot \vec{\text{n}}-\left(\left(\nabla \vec{\text{u}}\cdot \vec{\text{n}}\right)\cdot \vec{\text{n}}\right)\vec{\text{n}}\right\}, \end{array}$$

where $\nabla \vec{\text{u}}$ is the velocity gradient tensor, and t and n are the direction tangent and normal to the wall, respectively. The wall shear stress value is stored on the nearest Cartesian fluid cell to the wall, and in the post-processing, the value is projected onto the wall.

### Patient-specific cases

**Figure 7:**
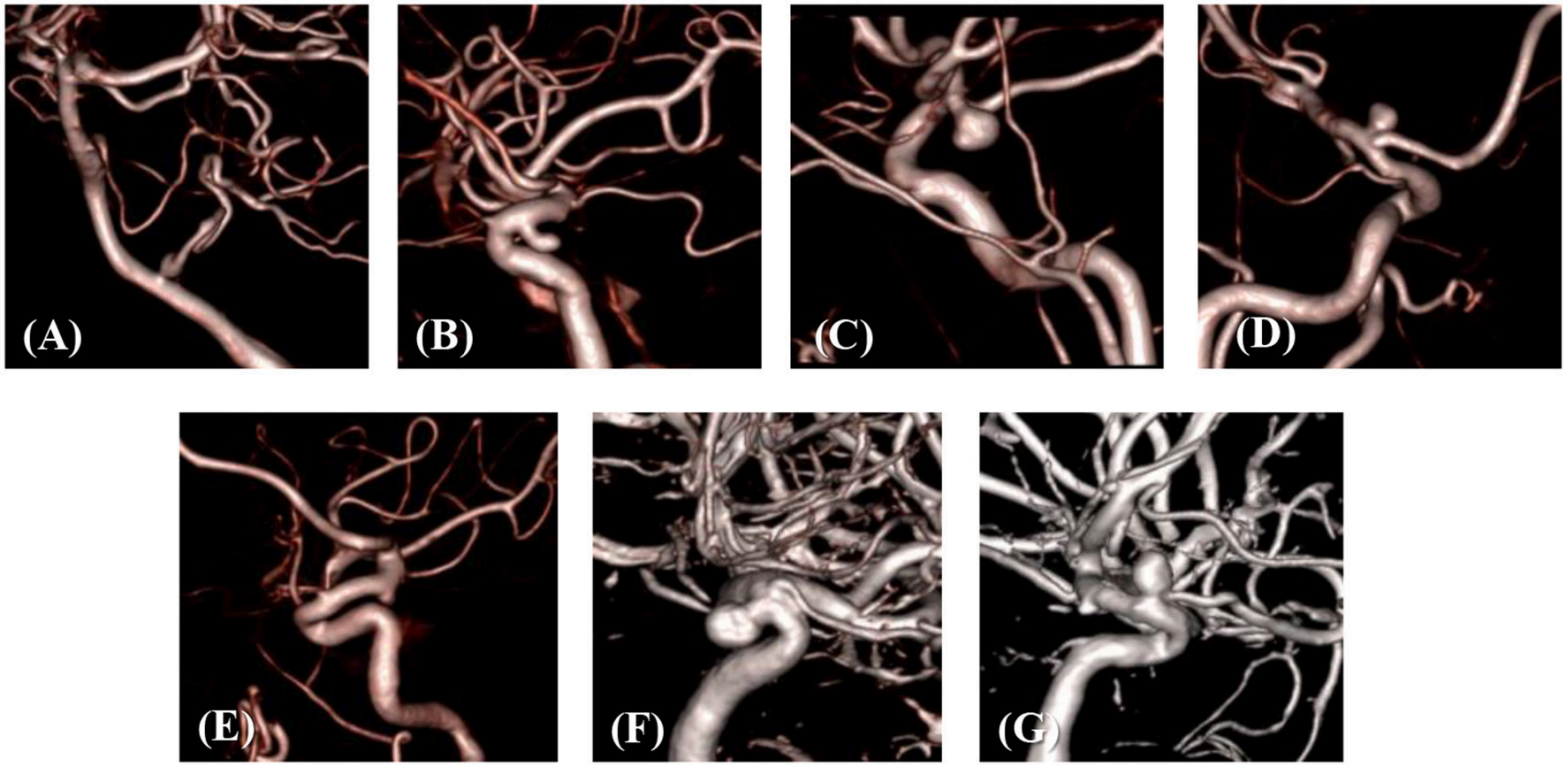
3D angiograms of patient-specific intracranial aneurysm cases **(A–G)**.

The developed simulation method is tested with patient-specific angiogram data. A total of seven anonymized patient-specific cases are selected from the Johns Hopkins University Intracranial Aneurysm Database (JHUIAD) so as to provide a range of aneurysm morphologies. The 3D angiograms for these cases are shown in Figure 7. The aneurysms are categorized into 3 types (fusiform, saccular, sidewall), and the size parameter, SR (size ratio: the ratio of aneurysm maximal length to the parent vessel diameter; Rahman et al., 2010) is listed in Table 1 for these cases.

**Table 1:** Types and size parameters for the patient-specific aneurysm cases.

**Case**	**A**	**B**	**C**	**D**	**E**	**F**	**G**
Type	Fusiform	Saccular	Saccular	Saccular	Saccular	Saccular	Saccular
Location	Branching	Sidewall	Bifurcation	Bifurcation	Sidewall	Sidewall	Sidewall
SR	1.28	1.5	1.87	1.33	0.77	1.29	1.7

*SR, the ratio of aneurysm maximal length to the parent vessel diameter*.

## Results

**Figure 8:**
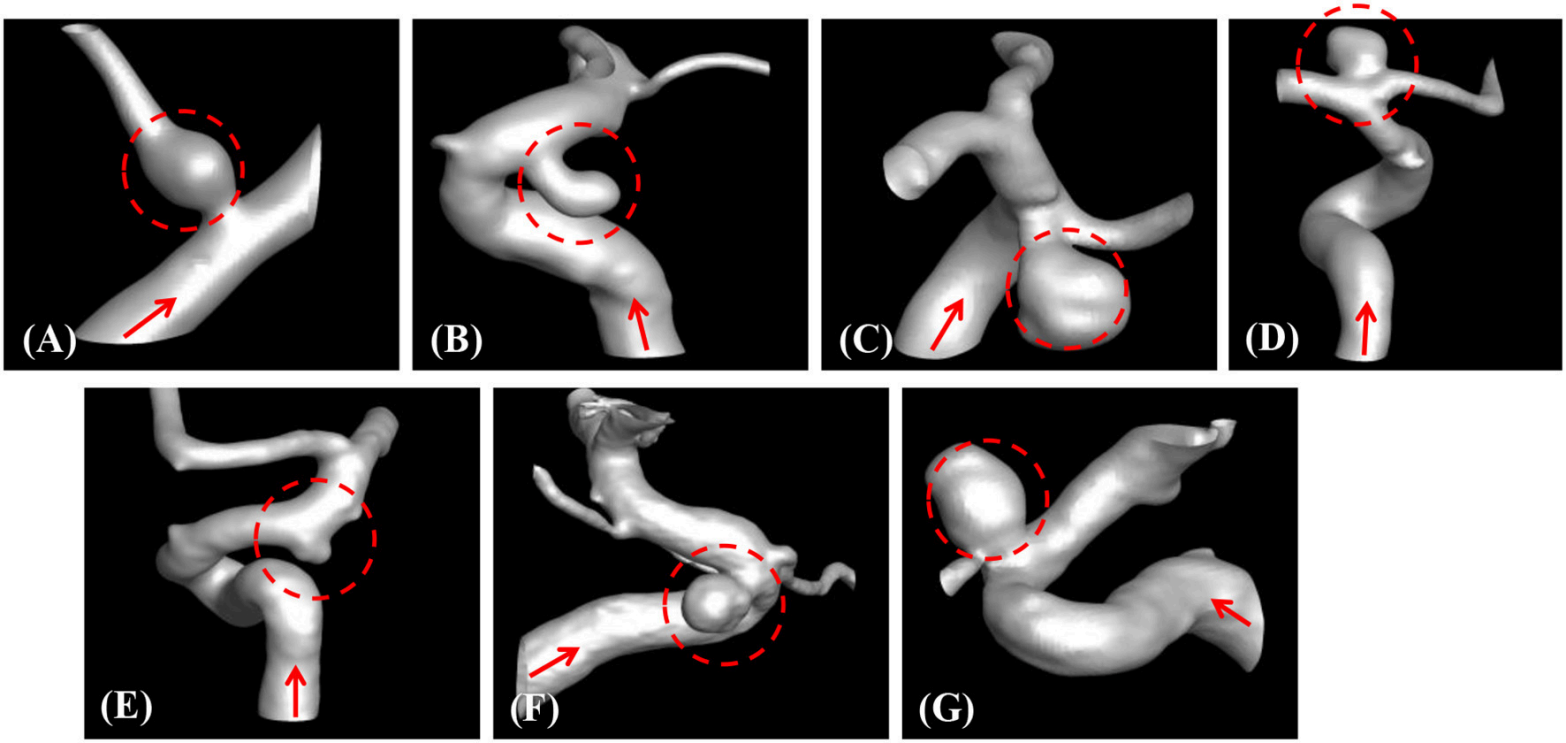
Automatically segmented aneurysm and vessel geometries using the present region-growing algorithm for the sample cases **(A–G)**. Target aneurysm and inflow direction are marked.

The developed method has been applied to the set of seven patient-specific cases shown in Figure 7. The cases include 1 fusiform (case A) at a branching, 2 saccular (cases C and D) type aneurysms located at bifurcation, and 4 sidewall saccular aneurysms (cases B,E–G). For the given angiogram data, an experienced neurosurgeon has performed the manual procedures described in section Region of Interest and Boundary Conditions. The neurosurgeon set the appropriate threshold intensity value and ROI, and provided the region growing seed point and the flow direction. The threshold intensity values are different for each case based on the overall contrast of the images, and chosen for the best representation of the morphology. The Cartesian ROI is determined to include sufficient length of the vessels both upstream and downstream from the target aneurysm. For cases with strong curvature of the vessel upstream of the aneurysm, the ROI is extended to include the upstream curved vessels. This is done so as to incorporate the effects of complex upstream flow on the aneurysm, and to minimize the artifacts due to the truncation of the domain. The lumen regions are then automatically identified by using the region-growing algorithm described in section Automatic Segmentation and Cleaning and the results are presented in Figure 8 for the sample cases. This shows that the present algorithm is capable of identifying the aneurysm and connected vessels for various types of the cerebral aneurysms.

**Figure 9:**
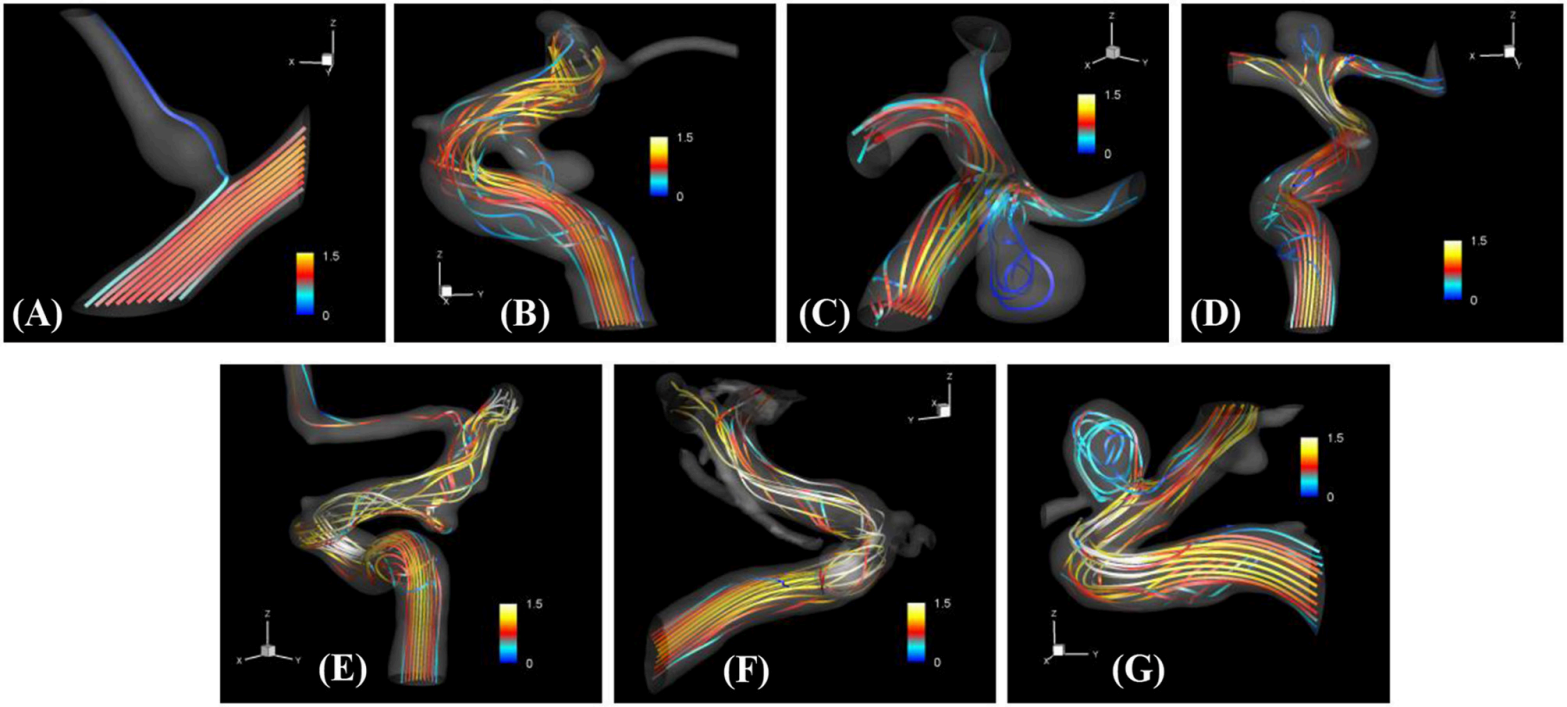
The results of automated hemodynamic simulations using the present immersed boundary, level-set method for the sample cases **(A–G)**. Streamtraces colored by the normalized velocity magnitude,$|\vec{u}|/U_{0}$. The color contours are truncated at the range shown in the color bar for the best visualization of local distributions.

**Figure 10:**
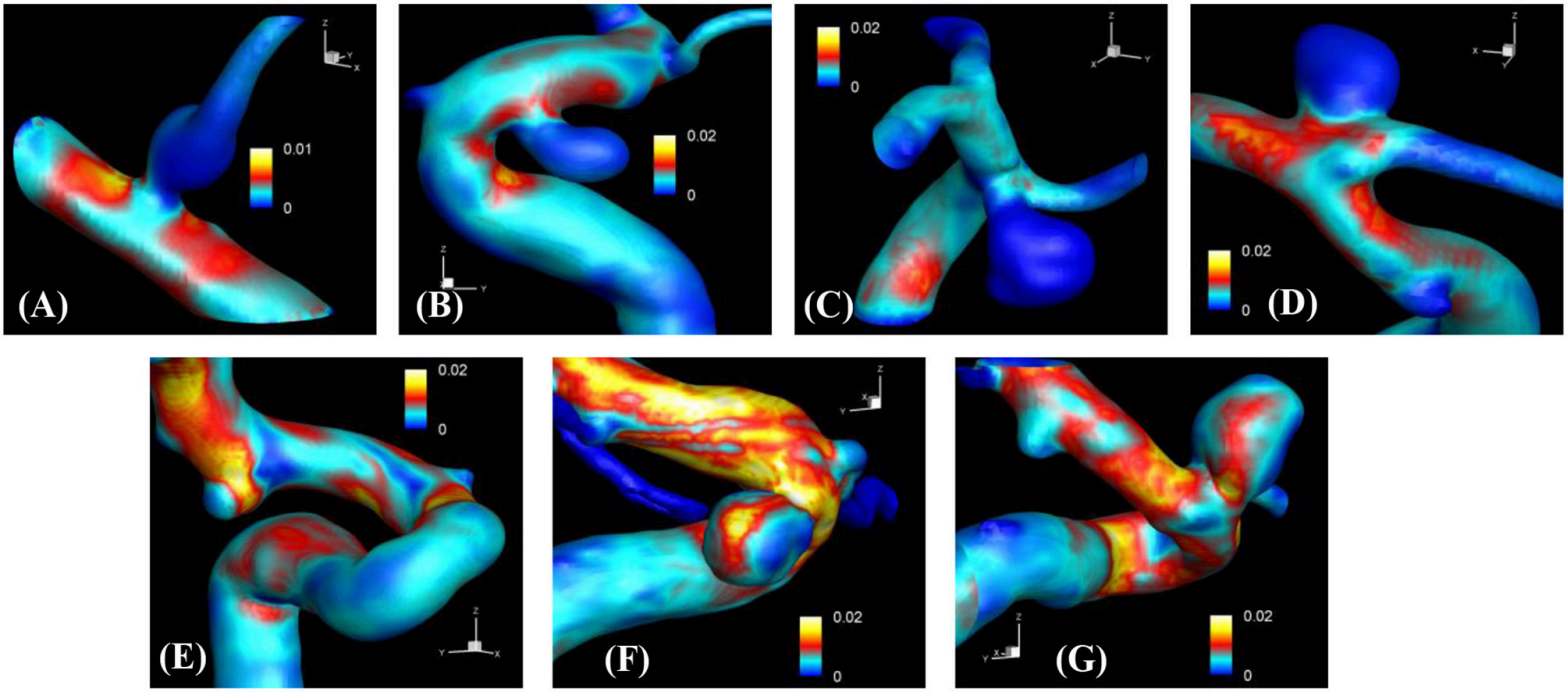
The results of automated hemodynamic simulations using the present immersed boundary, level-set method for the sample cases **(A–G)**. Iso-surfaces of ϕ = 0 are plotted and colored by the calculated magnitude of wall shear stress (unit in kPa). The color contours are truncated at the range shown in the color bar for the best visualization of local distributions.

The proposed simulation procedure and level-set based, immersed boundary flow solver have been applied to the prepared patient-specific aneurysm data shown in Figure 8. The computational domain covers the ROI and the 3D voxel space is directly used as the Cartesian grid for the flow simulations. The computation employed up to about 2 million Cartesian grid points with isotropic resolution of 0.2~0.27 mm depending on the ROI size and the voxel resolution. For the present flow simulations, a steady inflow velocity of 0.5 m/s, which is in the range of patient-specific blood flow speed reported in the previous study (Valencia et al., 2008), is applied to all cases. Flow simulations are performed for 5 s of real time which takes about 3 h. with 48 CPU cores on the MARCC (Maryland Advanced Research Computing Center) cluster for each case. The flow simulation results are presented in Figure 9, where the flow patterns are visualized via streamlines. Overall, the streamlines are tangent to the axial direction of the vessels, but as one can see in the figure, the curved vessels generate swirling flow patterns in the streamwise direction, i.e. streamwise vorticity (see Figures 9B,D–G). The wall shear stress (WSS) is then computed by the method described in section Post-Processing as a post-processing. The computed magnitude of WSS on the lumen wall surface is shown in Figure 10. Note that the boundary is not represented by the surface mesh but by the iso-contour of the level set function, ϕ = 0. Since the simulations are not performed with patient-specific inflow conditions, the overall magnitude of the WSS results may be outside the physiological range. Thus, only a comparative analysis of the WSS for the various cases is appropriate here. For most cases, the WSS magnitude is low on the aneurysm wall, and high on the aneurysm neck and walls of the parent vessel. For some cases however, (cases E–G), locally high WSS values are observed on the aneurysm wall. The results show that the present level-set based immersed boundary flow solver can resolve the hemodynamics for cerebral aneurysms with a wide range of shapes.

The hemodynamic metrics normalized suitably with the inflow velocity are listed in Table 2 for all cases. The WSS on the aneurysm wall is normalized by the inflow velocity, and its maximum (max), average over the aneurysm wall (avg), and variation (var) are calculated. In order to assess the overall flow strength inside the aneurysm, the normalized velocity magnitude is averaged over the volume inside the aneurysm and presented in Table 2 as well. The results are discussed in the following section.

**Table 2:** Normalized hemodynamic metrics.

**Case**	**A**	**B**	**C**	**D**	**E**	**F**	**G**
$\tau_{\text{w}}*,\text{max}$	0.0096	0.061	0.0075	0.031	0.062	0.11	0.09
$\tau_{\text{w}}*,\text{avg}$	0.0024	0.012	0.00062	0.0047	0.02	0.025	0.021
$\tau_{\text{w}}*,\text{var}$	0.0015	0.0081	0.00094	0.0039	0.012	0.017	0.013
${\left(|\vec{\text{u}}|/{\text{U}}_{0}\right)}_{\text{avg}}$	0.003	0.13	0.007	0.04	0.18	0.2	0.2

${\tau_{\text{w}}}^{*}=|{\vec{\tau }}_{\text{w}}|/\left(\rho {{\text{U}}_{0}}^{2}\right)$: wall shear stress normalized by inflow into the artery. Subscripts, max, avg, and var denote the maximum, average, and variation over the aneurysm wall, respectively. ${\left(|\vec{\text{u}}|/{\text{U}}_{0}\right)}_{\text{avg}}$: normalized magnitude of velocity averaged over the aneurysm volume.

## Discussion

In the present study, a highly-automated method to perform hemodynamic modeling of cerebral aneurysms using the patient-specific angiogram data has been proposed. The key idea is the direct use of voxelized contrast information from the 3D angiograms to construct a level-set function for the flow simulation with the immersed boundary method on a Cartesian grid. In this approach, the target aneurysm and vessels of interest can be segmented automatically, and no body-conformal surface/volume meshes need to be generated for the flow simulation. The Cartesian grid methods for the simulation of aneurysm hemodynamics were reported in the previous studies for the simple volume penalization method using the masking function (Mikhal and Geurts, 2014), the lattice Boltzmann method (He et al., 2009; Závodszky and Paál, 2013), and the boundary data immersion method (Otani et al., 2018). In the present study, we employ the masking function approach for the automatic segmentation of vessel/aneurysm from the medical imaging data, and the wall boundaries are represented by the level-set function constructed directly by using the image intensity information. For the simulation of aneurysm hemodynamics on the Cartesian grid, a well validated, “sharp-interface” immersed boundary method (Mittal et al., 2008) is adopted, and this solver can provide high resolution, high fidelity flow simulation results because the boundary conditions are imposed on the identified wall location precisely. By employing the present method, manual operations by a user to conduct hemodynamic simulations with the patient-specific angiogram data can be minimized, and this should in principle, enable us to scale up the hemodynamic modeling to very large number of sample cases.

The method developed in this study has been tested for a set of seven patient-specific cases picked from the Johns Hopkins Intracranial Aneurysm Database (JHUIAD). Although the sample size is in the current study is small, the cases involve a variety of aneurysm morphologies, sizes, and locations (see Figure 7 and Table 1). The developed algorithm successfully segmented various types of aneurysm and connected vessels within the ROI automatically as shown in Figure 8. The hemodynamic simulations for each case are then also performed automatically by the level-set based immersed boundary flow solver, and the results are presented in Figures 9, 10 and Table 2.

The present simulation results show that the values and the distribution of the wall shear stress (WSS) are very different for each patient-specific case. It should be noted that, although the peak WSS values in Figure 10 are in the range of reported values (Shojima et al., 2004), the current simulations are not performed with the patient-specific inflow conditions, and thus the WSS values could be over-predicted (McGah et al., 2014). Thus, comparative analysis of the WSS is warranted here. The present simulation results show that the aneurysms formed around the vessel branching/bifurcation (A,C,D) are exposed to low WSS in general. On the other hand, for the aneurysms on the sidewall of high curvature vessels (B,E–G), a higher WSS is observed, especially in the local region of the aneurysm wall. These observations are in-line with the previous computational studies (Castro et al., 2009; Valen-Sendstad and Steinman, 2014; Cebral et al., 2015). For bifurcation aneurysms, the flow inside the aneurysm is relatively weak (normalized average velocity magnitude: 0.003~0.04), and high values of WSS are observed only around the aneurysm neck and on the walls of the parent vessels (see Figures 10A,C,D; Shojima et al., 2004; Valencia et al., 2008; Castro et al., 2009). The WSS values on the aneurysm wall are consistently higher for the sidewall aneurysms as compared to ones at a bifurcation. This is because the high curvature of the vessel upstream the aneurysm results in more complex flow pattern and allows the stronger flow inside the aneurysm as one can see in Figures 9B,E–G. The locally high WSSs are observed at the location where the flow is impinging on or attaching to the aneurysm wall (Shojima et al., 2004; Castro et al., 2009; Cebral et al., 2015). The average velocity magnitude inside the aneurysm listed in Table 2 clearly shows this trend. The normalized average velocity magnitude for the sidewall aneurysms (0.13~0.2) are about an order-of-magnitude higher than the ones for the bifurcation aneurysms. The increase of flow strength and WSS for the saccular aneurysm on the sidewall of curved vessel was reported in the previous study (Meng et al., 2006). The present simulations show that the strong curvature of the upstream vessel can also affect the aneurysm hemodynamics (see Figures 9B,E,G). This implies that the ROI for the aneurysm hemodynamics simulation needs to be carefully chosen to include the effects of upstream vessel.

For all the cases (A–G), WSSs vary significantly over the range of an order-of-magnitude due to the different flow characteristics (normalized average WSS: 0.00062~0.025). For the present cases, the WSS is not correlated with the size parameter (SR). However, once the aneurysms are categorized by type or location, the WSSs in the same category show a similar order-of-magnitude. For example, the saccular aneurysms on the sidewall (cases B,E–G) present higher average WSS values (0.012~0.025), while the bifurcation aneurysms (A,C,D) show lower values (0.00062~0.0047). This suggests that a proper categorization of aneurysm morphology is essential for the reliable statistical analysis, and it also emphasizes the need for a large number of samples. Automation of the processes from patient imaging to hemodynamic modeling and post processing, such as is presented here would enable the scaling up of these models to very large sample sizes. Quantitative information regarding the hemodynamics of aneurysms obtained and analyzed for tens of thousands of cases could lead to fresh insights and new metrics regarding the factors that are responsible for aneurysm growth and rupture.

While the present study demonstrates that the method described here is capable of conducting simulations of aneurysm hemodynamics with very limited human intervention, the method has some limitations. First, there are still a significant number of user-defined features and actions such as the determination of segmentation criteria and the ROI size and the identification of inflow/outflow vessels, and these should be reduced to further automate the process. This could be accomplished by employing advanced image processing algorithms and methods such as machine learning. Second, the current method employs a fully developed inflow velocity profile, but if the upstream vessel has high curvature, a fully developed profile may not be valid. This issue could be addressed in a number of ways including by setting the ROI to avoid high curvature at the inflow boundary. For the outflow boundary condition, a traction-free condition is used in the present simulations. For more realistic hemodynamic modeling, the downstream boundary could employ a lumped-element model, which are quite well established in cardiovascular modeling (Esmaily-Moghadam et al., 2013; Min et al., 2015). Finally, in the present method, the voxel spacing of the angiogram data is directly used as a Cartesian grid spacing for the flow simulation. However, this grid resolution may not be enough especially for the smaller vessels. A simple resampling method based on the subdivision of the voxel can be employed to increase the Cartesian grid resolution for the flow simulation.

## Author contributions

J-HS and RM conception and design of research. JC and RT prepared and provided the data. J-HS and PE performed computations. J-HS and PE analyzed data. J-HS, PE, JC, and RM interpreted results of computations. J-HS prepared figures. J-HS and RM drafted manuscript. J-HS, RM, JC, and RT edited and revised manuscript. JHS, PE, RM, JC, and RT approved final version of manuscript.

### Conflict of interest statement

The authors declare that the research was conducted in the absence of any commercial or financial relationships that could be construed as a potential conflict of interest.
